# Automatic classification of uveal melanoma response patterns following ruthenium-106 plaque brachytherapy using ultrasound images and deep convolutional neural network

**DOI:** 10.1038/s41598-025-28995-3

**Published:** 2025-12-29

**Authors:** Atefeh Tahmasebzadeh, Elmira Yazdani, Masood Naseripour, Reza Mirshahi, Reza Ghaderi, Mahdi Sadeghi

**Affiliations:** 1 Medical Physics Department, School of Medicine, Iran University of Medical Sciences, Tehran, Iran; 2 Eye Research Center, The Five Senses Health Institute, Moheb Kowsar Hospital, Iran University of Medical Sciences, Tehran, Iran; 3 Finetech in Medicine Research Center, Iran University of Medical Sciences, Tehran, Iran; 4 Department of Electrical Engineering, Shahid Beheshti University, Tehran, Iran

**Keywords:** Uveal melanoma, Ruthenium-106, Ultrasound image, Response pattern, Multi-class classification, CNN, Eye cancer, Machine learning

## Abstract

Following uveal melanoma (UM) affected treatment using ruthenium-106 brachytherapy, tumor thickness patterns fall into one of four categories: decrease (regression), increase (recurrence), stop (stable), or other, which are assessed in follow-up A-mode and B-mode images. These patterns are critical indicators of the tumor’s response to therapy. This study aims to apply deep learning (DL) models for predicting post-brachytherapy tumor response patterns. A cohort of 192 patients participated in this study. B-Mode images taken at the time of diagnosis were collected, and the ophthalmologists labeled the images into four response patterns based on the results of the treatment. DenseNet121 and ResNet34 models were trained and evaluated using performance metrics. DenseNet121 achieved a macro-average AUC of 0.933 (0.95% CI [0.905–0.957]), compared to 0.916 (95%CI [0.884–0.945]) for the ResNet34. The per-class evaluation showed that DenseNet121 excelled in predicting all categories, providing superior predictive accuracy. This difference in classification performance was statistically significant based on the DeLong test (*p* < 0.05). The ablation study revealed that the best performance was achieved without pretrained weights, using dropout layers and a batch size of 32. Both models demonstrated strong classification capabilities, with DenseNet121 providing the highest overall accuracy. This study highlights the potential of DL models in predicting response patterns in UM patients undergoing brachytherapy. Further validation and exploration of their integration into clinical practice are warranted.

## Introduction

Uveal melanoma (UM) is the most prevalent and aggressive intraocular cancer, primarily affecting adults and frequently arising from the uveal tissue. Although new treatment methods such as proton therapy and gamma knife radiosurgery have emerged^1^, plaque brachytherapy, specifically with ruthenium-106 and iodine-125, remains the practical choice and preserves the globe. Numerous well-established factors, such as tumor size and thickness, location, retinal detachment, extrascleral extension, and retinal invasion, have been shown to influence anatomical outcomes^2,3^.

Tumor-specific genetic alterations and histopathologic characteristics, including epithelioid cell type, monosomy 3, 6p gain, and loss of the BAP-1 gene, are the key factors in predicting melanoma-specific patient outcomes^4^. Although fine-needle aspiration (FNA) can provide gene alteration results, it is often not available in any center^5^. Ultrasound (US) imaging, on the other hand, is widely available and essential for assessing the dimensions of UM and monitoring tumor status evaluation during follow-up^5^. Tumor response patterns, including thickness and basal diameter, are routinely evaluated from initial diagnosis to follow-up sessions using B-scan ultrasonography, and subretinal fluid resorption is assessed by fundoscopy^6^.

Radiotherapy initiates tumor response that is routinely evaluated in clinical practice as the change in tumor thickness measured by B-scan ultrasonography, and subretinal fluid resorption assessed by fundus photography (FPh)^7^. The response pattern is a critical indicator of the response to plaque therapy treatment. A few reports have highlighted instances of local treatment failure^7–10^, and subsequent enucleation, and others have indicated that rapid tumor regression following plaque brachytherapy is often associated with a poor survival prognosis^11,12^. Computer vision offers promising solutions to these challenges by assisting ophthalmologists in decision-making^13,14^, and existing models already incorporate clinical and demographic factors to predict individual patient prognosis following UM treatment^15–21^.

Recently, artificial intelligence (AI) has shown significant progress in big data retrieval, precise feature extraction, and improved consistency and efficiency in medical image analysis^22–26^. Deep learning (DL), a subset of AI, has become one of the most widely used approaches for medical image analysis, particularly in predicting cancers at early stages through convolutional neural networks (CNNs). This study represents an attempt to develop a DL-based predictive model for patients with UM following ruthenium-106 plaque brachytherapy, utilizing first and follow-up US images and clinical data. By investigating response pattern prediction through DL, this work aims to establish a clinically accessible model to enhance prognosis and guide treatment decisions, addressing a critical gap in personalized post-brachytherapy monitoring.

## Methods

### Study workflow

**Fig. 1:**
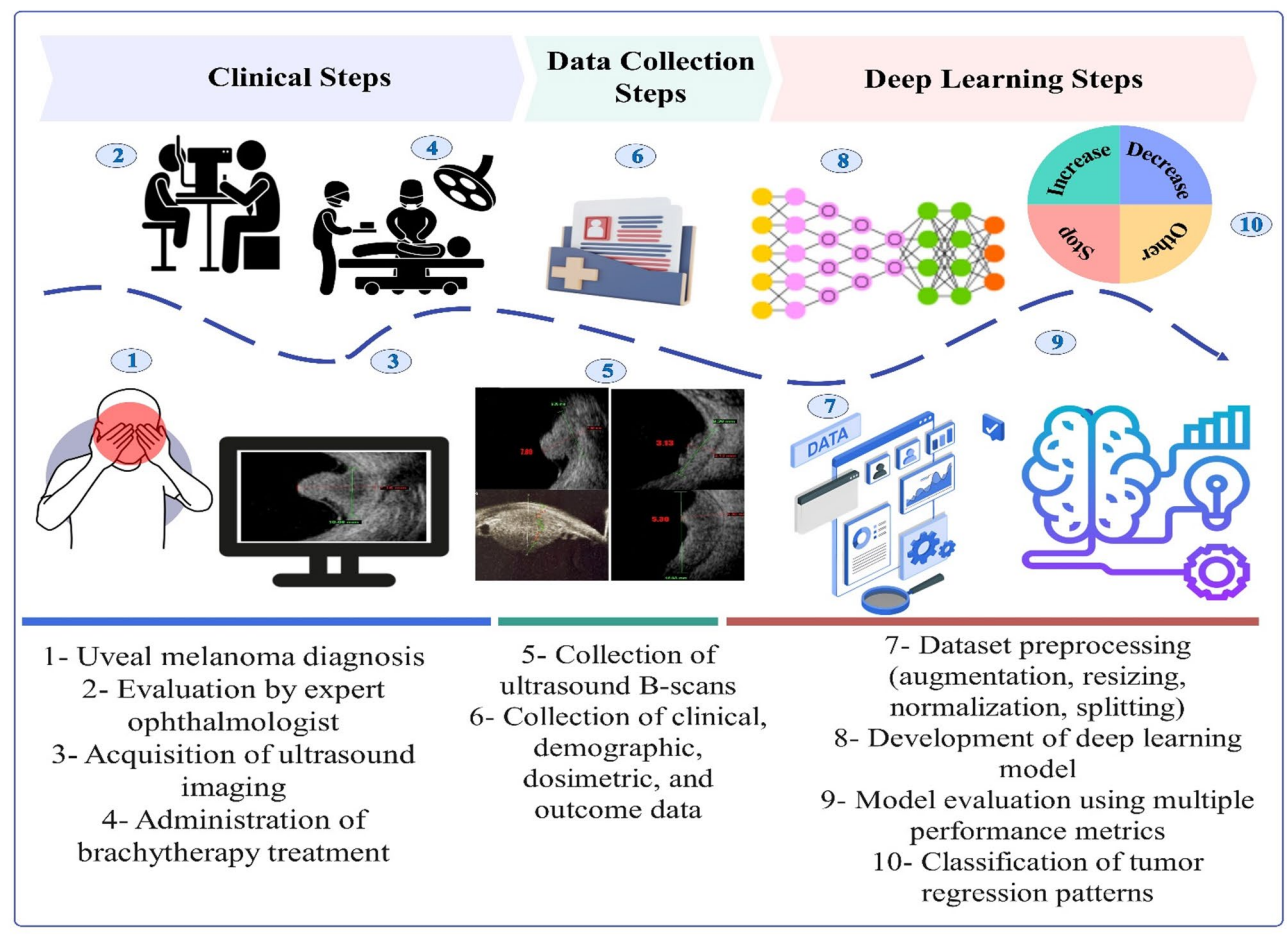
Workflow of the study illustrating the steps from UM diagnosis and plaque brachytherapy to deep learning model development. The process includes clinical examination, ultrasound imaging, data collection, preprocessing, and model evaluation, ultimately predicting UM response patterns.

Figure 1 illustrates the complete workflow adopted in this study, encompassing clinical procedures, data collection, and DL implementation. The objective is to explore the feasibility and effectiveness of DL in predicting UM response patterns. The process begins with the identification of UM in patients (Step 1), followed by a clinical examination by an Ocular oncologist (Step 2). Ultrasound imaging is performed (Step 3), and plaque brachytherapy is administered by a multidisciplinary team of physicians and medical physicists (Step 4). B-scan ultrasound images are then collected (Step 5), along with clinical, demographic, dosimetric, and follow-up data (Step 6). These data are preprocessed through augmentation, resizing, normalization, and dataset splitting (Step 7), after which the DL model is developed (Step 8) and evaluated using standard performance metrics (Step 9). The final outcome is a multi-class prediction of UM response patterns, including increase, decrease, stop, and other categories (Step 10).

### Study cohort

This retrospective, single-center study was conducted at the Eye Research Center. Due to the retrospective nature of the study, the Ethics Committee of IUMS (IR.IUMS.FMD. REC.1402.417) waived the need to obtain informed consent and approved the experiments. All methods were performed in accordance with the relevant guidelines and regulations. The study population included adult patients clinically diagnosed with UM between July 2017 and 2022 who underwent treatment with ruthenium-106 plaque brachytherapy. The exclusion criteria included: (1) age under 18, (2) prior treatment with therapies other than ruthenium-106 plaque brachytherapy, (3) follow-up duration of less than two years, (4) presence of metastatic disease at diagnosis, and (5) patients of other nationalities.

B-Mode ultrasound images of 192 patients were collected at the time of uveal melanoma diagnosis and before the start of brachytherapy treatment. Each patient had at least two images in both transverse and longitudinal views at the time of diagnosis, so a total of 661 images were collected. Each patient had at least 2 years of follow-up, so the ocular oncologist labeled the patients’ images into four groups in terms of response pattern, increase, decrease, stop, and other, based on the response to treatment of the mass during follow-up sessions. Additionally, US images were independently interpreted and reported by two board-certified ophthalmologists specializing in ocular oncology, each with a minimum of 10 years of experience.

### Response patterns in intraocular melanoma post-brachytherapy

**Fig. 2:**
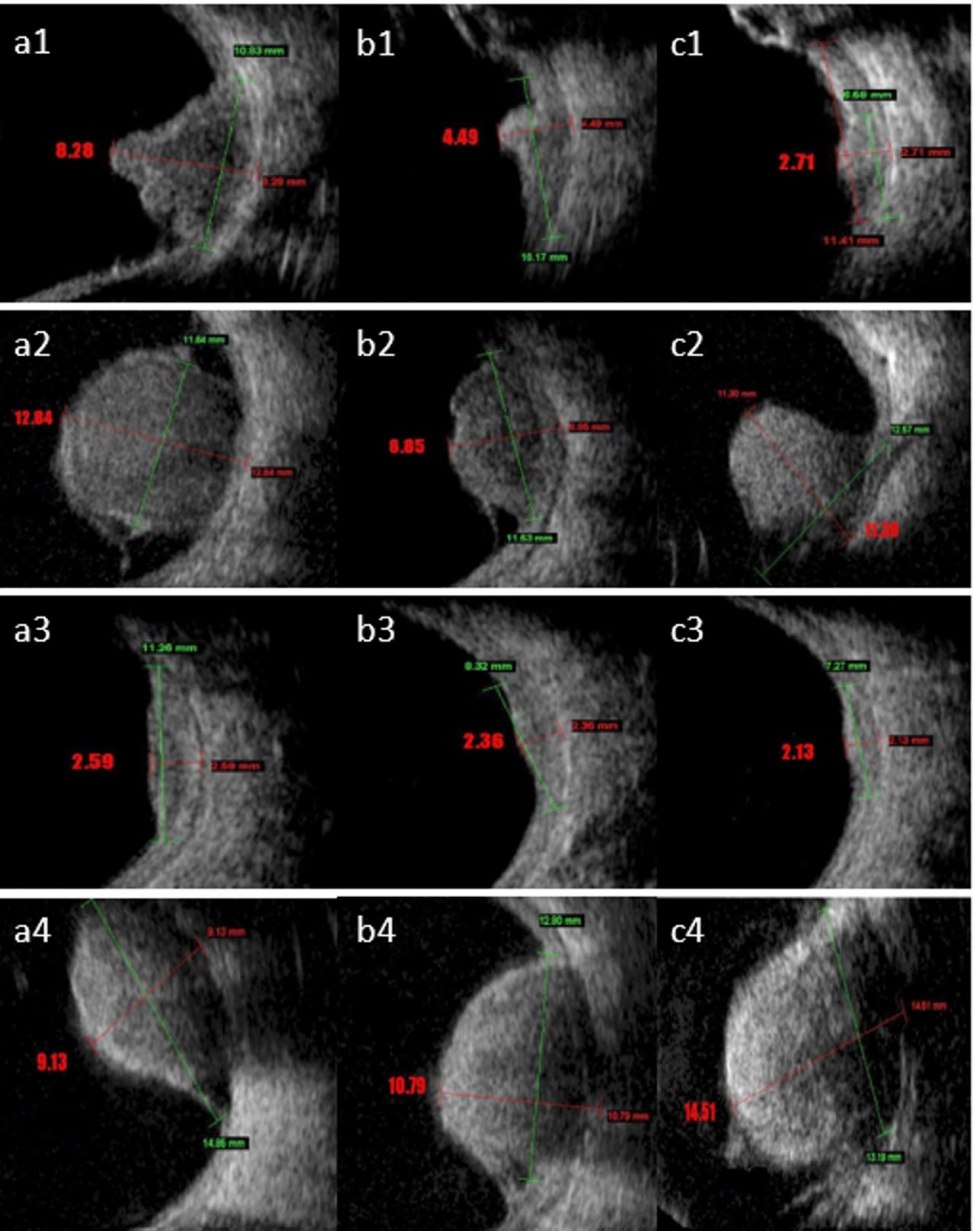
B-Mode ultrasound images of four different patients with Decrease, Other, Stop, and Increase response pattern at the time of diagnosis (a1, a2, a3, and a4), one year (b1, b2, b3, and b4), and two years (c1, c2, c3, and c4) after treatment with Ru-106 plaque brachytherapy.

Patients were treated with ruthenium 106 (Eckert & Ziegler BEBIG company, Berlin, Germany) plaques to deliver the appropriate dose to the apex and base of the tumor. Three plaque types were utilized: round (CCA, CCB, and CGD), notch (COB), and Ciliary body (CIA). An experienced medical physicist oversaw the dosimetry process, ensuring accurate dose delivery based on the activity and half-life of the ruthenium-106 plaques. The physicist also documented the dosimetry reports for each treatment. An ocular oncologist measured tumor thickness and the largest basal diameter (LBD). Tumor thickness was assessed from the inner scleral surface to the tumor apex along two meridians: one along the LBD and another perpendicular to it. Representative digitized scans were prospectively stored during each diagnostic and follow-up visit. We tracked the changes in tumor thickness and the LBD over time and classified tumor response patterns according to Abramson et al.^27^, using four main categories: D (decrease; a progressive reduction in thickness by at least 15% post-brachytherapy), S (stable; less than 15% change in thickness), I (increase; a progressive increase in thickness by at least 15%), and Others^27^. The Others category was further divided into five subtypes: DS (decrease followed by stability), DI (decrease followed by increase), ID (increase followed by decrease), SD (stability followed by decrease), and zigzag (alternating measurements with no clear trend). A total of 661 images were collected from 192 patients with intraocular melanoma before the beginning of treatment. All images were acquired by a single operator and independently interpreted by two ophthalmologists, each with over ten years of experience in plaque radiotherapy for UM. As illustrated in Fig. 2, B-Mode US images of four different patients with Decrease, Other, Stop, and Increase response pattern at the time of diagnosis (a1, a2, a3, and a4), one year (b1, b2, b3, and b4), and two years (c1, c2, c3, and c4) post-treatment with Ru-106 plaque brachytherapy.

### Data preprocessing

All US images were obtained in JPEG format from the US imaging databases. A 5-fold cross-validation (CV) approach was applied to split the dataset, using 80% for training and 20% for testing in each fold. To address the class imbalance, oversampling was performed by applying horizontal flipping, rotation, shifting, scaling, and brightness/contrast adjustments. This process ensured that all classes contained equal number of images.

Before augmentation, the Increase group contained 36 images, the Decrease group had 336 images, the Stop group included 125 images, and the Other group consisted of 164 images, and after augmentation increased to 3966 images. Before model training, images were resized to 224 × 224 pixels to match input requirements. Normalization was applied using a mean of [0.485, 0.456, 0.406] and a standard deviation of [0.229, 0.224, 0.225] and conversion to tensor format, ensuring consistency with pre-trained models.

### Deep learning model construction

Two convolutional neural network architectures, DenseNet121 and ResNet34, were utilized as the backbone models for image multi-class classification. These architectures were chosen due to their proven ability to perform well in image classification tasks while maintaining reasonable computational efficiency. Both models were initialized with random weights (weights = None) and trained under identical conditions to ensure a fair comparison.

DenseNet121 is composed of five dense blocks organized across three transition levels, with each block containing multiple convolutional layers followed by a max-pooling operation. The architecture leverages dense connectivity, where each layer receives input from all preceding layers, promoting feature reuse and mitigating the vanishing gradient problem. The final output, after global average pooling, is passed through a series of fully connected layers, followed by a dropout layer (*p* = 0.4) tailored for four-class classification.

ResNet34, on the other hand, is structured around residual learning, consisting of an initial convolutional layer followed by four stages of residual blocks, each incorporating identity shortcut connections. These skip connections allow gradients to propagate more effectively during training, enabling deeper network architectures without degradation in performance. In the case of ResNet34, the final fully connected layer was similarly modified to include a linear transformation and a dropout layer with the same probability of performing four-class classification. Both models were trained using 2D ultrasound images and the corresponding ground truth labels provided in the dataset. Input images were resized to 224 × 224 pixels and normalized using ImageNet statistics.

Training was performed using stochastic gradient descent (SGD) with a learning rate of 0.001, momentum of 0.9, and weight decay of 1e-4. The loss function used was cross-entropy with label smoothing (ε = 0.1). A five-fold CV approach was implemented to ensure robustness. Training was conducted over 200 epochs, where each epoch involved forward propagation, loss computation, gradient backpropagation, and weight updates.

### Ablation study

To evaluate the contribution of individual components within the model architecture and training strategy, a series of ablation experiments were performed. Each experiment involved modifying a specific element while keeping all other parameters constant to isolate its impact on model performance. Since DenseNet121 outperformed ResNet34, all three ablation scenarios were conducted exclusively for DenseNet121: (1) initializing the network with pre-trained ImageNet weights, (2) reducing the batch size to 16, and (3) removing dropout layers from the architecture. The results of these experiments provided insights into the sensitivity of the model to various training configurations and optimization strategies.

### Performance evaluation

The model performance is validated on the test set after each epoch, with key performance metrics logged for analysis. These metrics provide a comprehensive assessment of the model’s performance:

- Confusion Matrix, Accuracy, Recall (Sensitivity), Precision, F1 Score, ROC Curve, and AUC (area under the curve).
- Macro average computes the AUC for each class individually and then averages the results. This approach treats all classes equally, regardless of their frequency in the dataset.
- Micro average aggregates the contributions of all classes to compute a global AUC score.

These averages provide a broader view of model performance across all classes.

- Cohen’s Kappa Coefficient: The Kappa coefficient evaluates agreement between predicted and actual classifications while accounting for chance agreement. It ranges from − 1 to 1, where a Kappa of 1 indicates perfect agreement, 0 suggests no agreement beyond chance, and values below 0 reflect worse-than-random performance, indicating poor model reliability. P-values < 0.05 were considered statistically significant.

These metrics together offer a robust evaluation of model performance across multiple dimensions. To estimate the statistical uncertainty associated with each metric, 95% confidence intervals (CIs) were calculated using bootstrap resampling with 1,000 iterations. In each iteration, the test set was resampled with replacement, and metrics such as accuracy, F1-score, and AUC were recalculated. The resulting metric distributions were then used to derive percentile-based 95% CIs. To assess whether the differences in AUC between models were statistically significant, DeLong’s test was applied.

### Ethics approval

The Ethics Committee of IUMS waived the requirement for ethical approval (IR.IUMS.FMD. REC.1402.417).

## Results

### Baseline characteristics

A total of 192 patients with UM treated with plaque brachytherapy were included in this study. The cohort comprised 60.5% females and 39.5% males, with choroidal melanoma being the most common type (63%) and ciliary body involvement in 37%. Tumor sizes were evenly distributed between small (26%), medium (26%), and large (35.5%), with 12.5% classified as very large. Most of the tumors were dome-shaped (74%) and located in the temporal part (28%). The most common inserted plaques were CGD (34.9%) and CCB (28.1%). The right eye was affected in 52.1% of cases. Diabetes was present in 17.2% of patients. The mean age was 52.5 ± 14.1 years, and the apex and scleral doses averaged 85.5 ± 2.5 Gy and 906.1 ± 478.6 Gy, respectively, with a mean radiation time of 116.9 ± 92.3 h. Also, the response patterns after plaque radiotherapy showed 4.8%, 51.5%, 27.2%, and 16.5% increase, decrease, other, and stop, respectively.

### Model evaluation

To assess the overall classification performance of the two models, DenseNet121 and ResNet34, a five-fold CV approach was employed. The averaged training and validation metrics across folds are summarized in Table 1. This table provides a comprehensive view of each model’s global performance, not per class, including accuracy, precision, recall, F1 score, and Cohen’s Kappa. These metrics reflect how well the models generalize across different subsets of the data and serve as a benchmark for overall model robustness.

**Table 1:** Mean performance metrics across 5-fold cross-validation for ResNet34 and DenseNet121 models.

Metrics	Dataset	ResNet34	DenseNet121
Accuracy	Train	0.81	*0.83*
	Validation	0.77	*0.80*
Precision	Train	*0.85*	0.84
	Validation	0.74	*0.77*
Recall	Train	0.81	*0.82*
	Validation	0.73	*0.76*
F1 Score	Train	*0.81*	*0.81*
	Validation	0.72	*0.76*
Kappa (*p* < 0.05)	Train	0.71	*0.78*
	Validation	0.60	*0.63*

To further understand model behavior across specific output categories, we conducted a per-class analysis. The performance metrics for each class, Decrease, Increase, Other, and Stop, are detailed separately for DenseNet121 and ResNet34 in Tables 2 and 3, respectively. These tables report the class-wise training and validation accuracy, precision, recall, and F1 scores, based on the epoch where each class achieved its highest validation accuracy. This per-class breakdown provides insights into model strengths and potential limitations when distinguishing among specific therapeutic response categories.

**Table 2:** Per-class mean performance metrics for the DenseNet121 model.

Category	Train Accuracy	Val Accuracy	Train Precision	Val Precision	Train Recall	Val Recall	Train F1 Score	Val F1 Score
Decrease	0.88	0.87	0.87	0.83	0.92	0.87	0.87	0.85
Increase	0.97	0.91	0.90	0.87	0.91	0.88	0.90	0.88
Other	0.92	0.88	0.92	0.86	0.86	0.81	0.88	0.82
Stop	0.93	0.92	0.95	0.93	0.86	0.84	0.89	0.88
Mean	0.92	0.89	0.91	0.87	0.89	0.85	0.88	0.86

**Table 3:** Per-class mean performance metrics for the ResNet34 model.

Category	Train Accuracy	Val Accuracy	Train Precision	Val Precision	Train Recall	Val Recall	Train F1 Score	Val F1 Score
Decrease	0.87	0.85	0.85	0.83	0.91	0.85	0.85	0.85
Increase	0.97	0.91	0.90	0.88	0.88	0.78	0.88	0.82
Other	0.92	0.85	0.92	0.81	0.85	0.81	0.88	0.80
Stop	0.90	0.91	0.96	0.89	0.89	0.84	0.88	0.87
Mean	0.915	0.88	0.90	0.85	0.88	0.82	0.87	0.83

For a more focused comparison of validation performance between the two models, we summarized the key metrics side by side in Table 4. This comparative table highlights the differences in classification ability between DenseNet121 and ResNet34 for each class on the validation set. It includes metrics such as validation accuracy, precision, recall, F1 score, and AUC, allowing a nuanced evaluation of which model performs better for each therapeutic response type. This analysis is particularly valuable for identifying model-specific strengths and for guiding the choice of architecture in clinical decision support contexts. To further validate these findings, DeLong’s test was applied to assess the statistical significance of AUC differences between the two models (*p* < 0.05). The results confirmed that the higher AUC values achieved by DenseNet121 were statistically significant across classes, indicating superior discriminative performance compared to ResNet34. This strengthens the case for selecting DenseNet121 in settings where reliable classification of response patterns is critical.

**Table 4:** Mean performance (± 95% confidence interval) of DenseNet121 and ResNet34 on the validation set across response classes.

Class/ Model	Accuracy		Precision		Recall		F1 Score		AUC	
	ResNet34	DenseNet121	ResNet34	DenseNet121	ResNet34	DenseNet121	ResNet34	DenseNet121	ResNet34	DenseNet121
Decrease	0.85 (0.82–0.88)	0.87 (0.84–0.90)	0.83 (0.79–0.86)	0.83 (0.80–0.86)	0.85 (0.81–0.88)	0.87 (0.84–0.89)	0.85 (0.81–0.87)	0.85 (0.82–0.87)	0.89 (0.86–0.92)	0.90 (0.87–0.93)
Increase	0.91 (0.89–0.94)	0.91 (0.89–0.93)	0.88 (0.85–0.91)	0.87 (0.84–0.89)	0.78 (0.75–0.82)	0.88 (0.85–0.91)	0.82 (0.79–0.85)	0.88 (0.85–0.90)	0.91 (0.88–0.94)	0.90 (0.87–0.93)
Other	0.85 (0.81–0.88)	0.88 (0.85–0.91)	0.81 (0.77–0.85)	0.86 (0.82–0.89)	0.81 (0.77–0.85)	0.81 (0.77–0.85)	0.80 (0.76–0.83)	0.82 (0.78–0.86)	0.87 (0.83–0.91)	0.88 (0.84–0.92)
Stop	0.91 (0.88–0.94)	0.92 (0.89–0.94)	0.89 (0.86–0.92)	0.93 (0.90–0.96)	0.84 (0.80–0.87)	0.84 (0.81–0.88)	0.87 (0.83–0.90)	0.88 (0.85–0.91)	0.93 (0.90–0.96)	0.95 (0.92–0.97)
Mean	0.88 (0.86–0.91)	*0.89* (0.87–0.92)	0.85 *(0.82–0.88)*	*0.87 (0.84–0.89)*	0.82 (0.79–0.85)	*0.85 (0.82–0.88)*	0.83 (0.80–0.86)	*0.86 (0.84–0.89)*	0.89 (0.87–0.93)	*0.90 (0.88–0.94)*

**Fig. 3:**
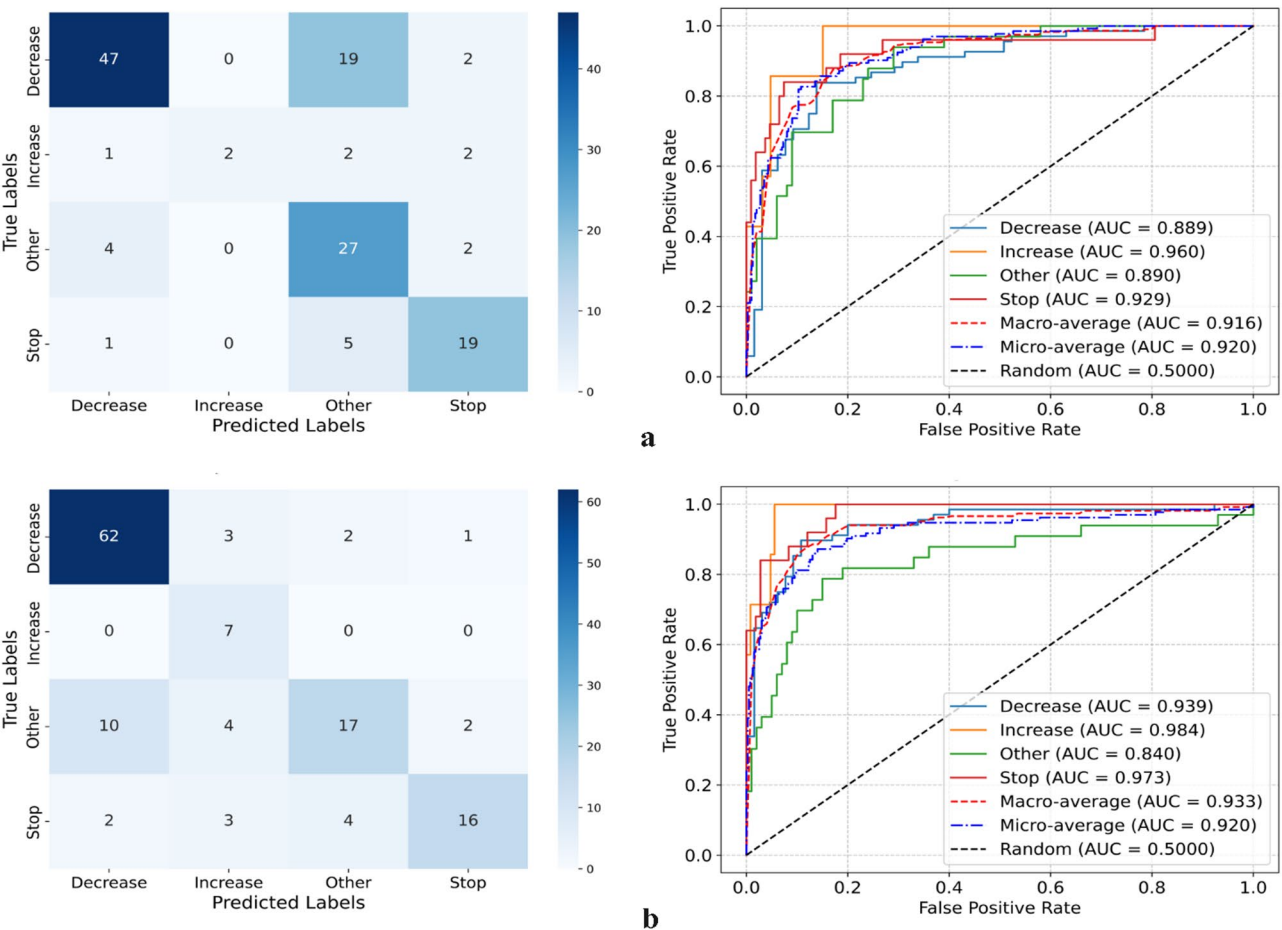
Model performance on the validation set from fold 0 (randomly selected). (a) ResNet34 and (b) DenseNet121, showing confusion matrices and ROC curves for each model.

Figure 3 illustrates the classification performance of the two evaluated models, ResNet34 and DenseNet121, on the validation set from fold 0. Panel (a) shows that ResNet34 achieved strong class separation, particularly for the Stop and Other classes, although some confusion remained between Decrease and Other. The corresponding ROC curves reveal high AUC values across all classes, with a macro-average AUC of 0.916 (95%CI [0.884–0.945]). In comparison, panel (b) demonstrates that DenseNet121 achieved superior performance, correctly classifying a larger number of Decrease and Increase samples, as reflected in its confusion matrix. The ROC analysis further supports this observation, with DenseNet121 reaching higher AUCs, including 0.984 for Increase and a macro-average AUC of 0.933 (0.95%CI [0.905–0.957]). The difference in macro-average AUCs between the two models was statistically significant based on the DeLong test (*p* < 0.05), confirming the improved performance of DenseNet121.

### Ablation study

**Fig. 4:**
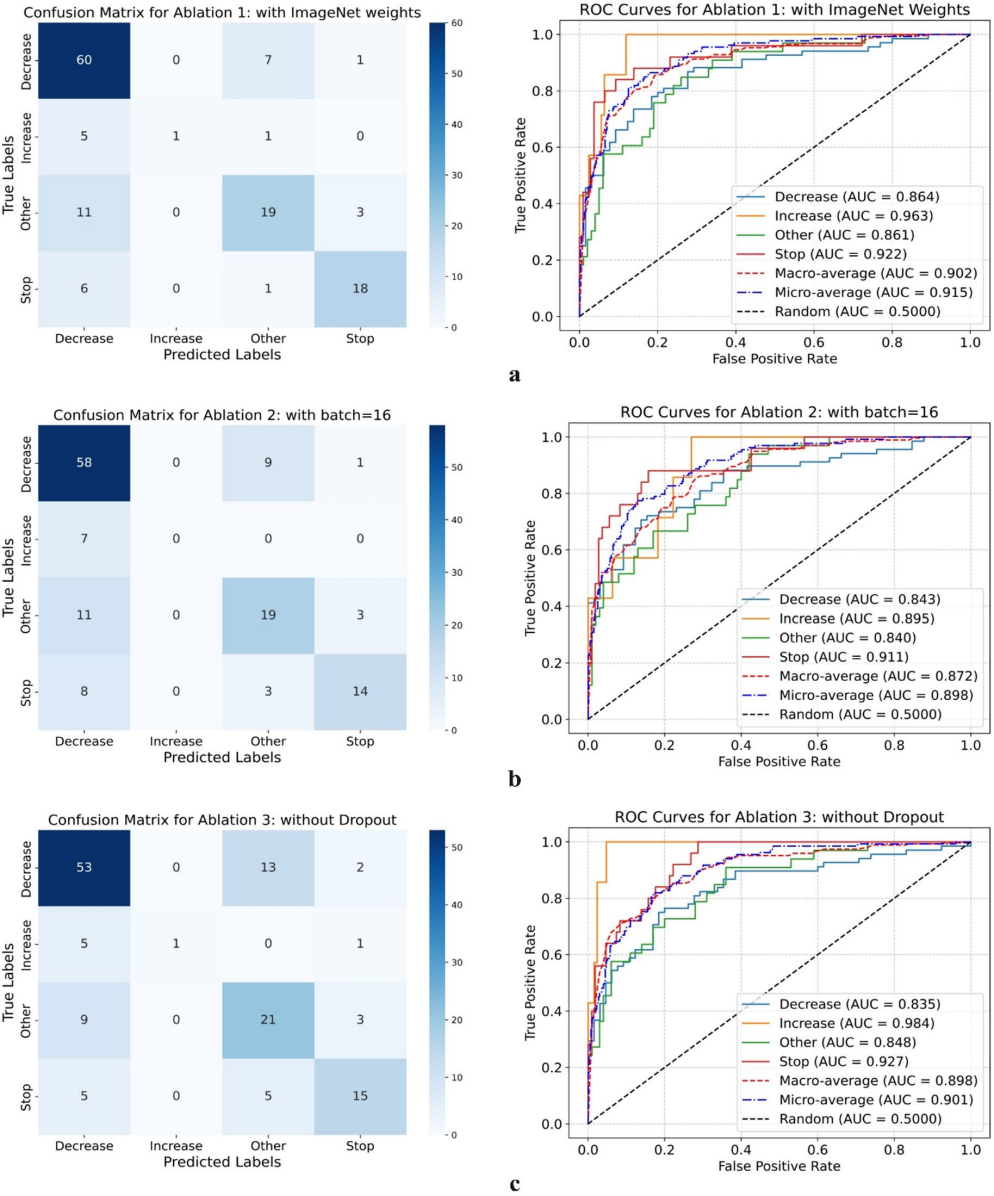
Confusion matrices and ROC curves from fold 0 (randomly selected) for the three ablation scenarios using the DenseNet-121 model: (a) with pretrained ImageNet weights; (b) reduced batch size (16); (c) without dropout.

Table 5 provides detailed validation metrics, including accuracy, precision, recall, F1-score, and AUC for each scenario across all classes. The final column corresponds to the baseline configuration, included for comparison with the other three scenarios, and it is evident that it achieves the best overall performance among all. Additionally, Fig. 4 (a to c) presents the confusion matrices and ROC curves for each ablation scenario (1–3), corresponding to subfigures (a) through (c), respectively. The results demonstrate that the baseline configuration employing dropout layers, no pretrained weights, and a batch size of 32 outperformed all other ablation variants. Specifically, deviations from this setup, such as removing dropout, incorporating pretrained weights, or reducing batch size, resulted in reduced classification performance. These findings highlight the robustness and effectiveness of the main training configuration for DenseNet121 in this context.

**Table 5:** Mean validation metrics for the three ablation scenarios across all classes using the DenseNet121 model. The final column corresponds to the baseline configuration (with dropout, no pretrained weights, batch size of 32), which achieved the best overall performance.

Class	Metrics\Scenarios	1: With pretrained ImageNet weights	2: With a batch size of 16	3: Without dropout	Main
Decrease	Accuracy	0.82	0.76	0.78	*0.87*
	Precision	0.83	0.78	0.79	*0.85*
	Recall	0.82	0.76	0.78	*0.87*
	F1 Score	0.80	0.76	0.78	*0.85*
	AUC	0.88	0.84	0.83	*0.90*
Increase	Accuracy	*0.97*	*0.97*	0.95	0.91
	Precision	0.95	*0.98*	0.70	*0.98*
	Recall	0.76	0.71	0.59	*0.88*
	F1 Score	0.80	0.79	0.61	*0.88*
	AUC	0.92	0.89	*0.98*	0.90
Other	Accuracy	0.86	0.82	0.84	*0.88*
	Precision	*0.84*	*0.77*	0.82	0.75
	Recall	0.78	0.73	0.74	*0.81*
	F1 Score	0.80	0.75	0.76	*0.82*
	AUC	*0.88*	0.84	0.84	*0.88*
Stop	Accuracy	0.90	0.91	0.89	*0.92*
	Precision	0.86	*0.89*	0.86	*0.87*
	Recall	0.80	0.82	0.78	*0.84*
	F1 Score	0.83	0.85	0.80	*0.88*
	AUC	0.92	0.91	0.92	*0.95*

## Discussion

Recent advancements in AI have significantly transformed medicine, particularly in the analysis of large datasets, enabling more accurate diagnostic and prognostic predictions. Numerous studies have highlighted the potential of computer-aided systems in enhancing clinicians’ diagnostic capabilities^28,29^. Understanding prognosis, both pre- and post-surgery, is crucial for patients, reducing anxiety and enabling future planning^30,31^. Deep learning models offer efficient and accurate predictions and classifications for diverse diseases using imaging data, including breast cancer^28^, liver diseases^29^, colon cancer^30^, brain tumor^31^, skin cancer^32^, lung cancer^33^, pneumonia^34^, and COVID-19^34^, all with minimal human oversight.

In terms of classifying, diagnosing, and prognosing UM, previous studies have applied a combination of different imaging modalities, including US, fundus photography, and optical coherence tomography (OCT), along with AI, to improve accuracy and predictive performance^35^. Notably, this study represents the first attempt to use deep learning models to predict the response pattern of UM using pre-treatment ultrasound images, which are labeled by ocular oncologists based on the results of treatments. By addressing this gap, our work provides a novel approach to personalizing treatment plans for UM patients and enhancing the predictive power of clinical decision-making.

Previous AI-based studies in UM have leveraged different imaging techniques to classify and diagnose ocular melanoma. For example, Biswarup et al.^36^ developed a CNN-based method for detecting ocular melanoma using fundus photography images from 170 pre-melanoma patients, while Zhang et al.^37^ explored the correlation between iris color and UM. AI, combined with image modalities, can also differentiate UM from nevus and other lesions. In this regard, Ahmed et al.^38^ employed the US images and an artificial neural network (ANN) approach for intraocular melanoma classification. Yao et al.^39^ applied DenseNet121 and FPh to classify uveal melanoma and choroidal nevi, and examined the color fusion effect on UM and uveal nevus classification, and Dadzie et al.^40^ applied DenseNet121 and FPh to an automated diagnosis of UM. Goswami et al.^41^ evaluated DL models for distinguishing benign from malignant intraocular tumors, and finally, Sabazade et al.^42^ developed a DL model to differentiate small choroidal melanomas from nevi, while Hoffmann et al.^43^ evaluated DL for classifying choroidal melanocytic lesions based on FPh images. Valmaggia et al.^44^ assessed OCT-based auto-segmentation of pigmented choroidal lesions (PCLs) using DL. Additionally, Jiechao Ma et al.^45^ employed ultra-wide-field fundus photography images and the DeepLabv3 architecture for UM segmentation, presenting a model with high sensitivity for the early diagnosis of UM tumors. Despite these advancements, no study has yet attempted to predict UM’s response pattern using pre-treatment ultrasound images, an area this study pioneers, ultimately contributing to more accurate prognosis and tailored therapeutic strategies for patients.

This study specifically characterizes the post-brachytherapy response patterns of UM by analyzing initial tumor thickness and LBD, which emerged as powerful and valuable predictors of treatment outcomes. Abramson et al.^27^ analyzed 82 patients to assess tumor response patterns, reporting that no two UMs regressed identically after brachytherapy. In their study, 70% of cases exhibited progressive regression (pattern D), 16% remained stable (pattern S), 12% increased in size (pattern I), and 2% followed other response patterns (pattern O). In contrast, our study identified 4.8% of UM increased over time, with 51.5% following pattern D, 27.2% pattern O, and 16.5% pattern S. Rui Fang et al.^46^ found that 36% of UMs remained stable, while 46.8% exhibited pattern D regression, similar to the results of Rashid et al.^47^. A possible explanation for the differences observed could lie in the use of different isotopes, as the type of isotope has been reported as an independent predictor of tumor regression^48^. The Other response pattern in our study encompassed five distinct subtypes (DS, DI, ID, SD, and zigzag), accounting for 27.2% of cases, higher than previously reported. This discrepancy likely reflects longer follow-up and isotope-related differences in tumor regression response. Combining these subtypes into one class was necessary to ensure sufficient data for model training; therefore, due to variations in treatment planning strategies among ophthalmic oncology centers and the use of different radioisotopes, the observed response patterns are not expected to be completely identical across institutions^49^. Additionally, the tumor’s original height was positively associated with regression rate, aligning with the findings of Rashid et al^48^., which indicated that larger tumors tend to shrink significantly faster than smaller ones. Future research with larger multi-center cohorts could enable separate analysis of these subtypes or employ hierarchical models to improve classification granularity and clinical relevance.

Two CNN architectures, DenseNet121 and ResNet34, were evaluated for multi-class classification of uveal melanoma response patterns using pre-treatment ultrasound images. After comparing several deep networks, these two models showed the most stable learning behavior with minimal overfitting.

Using five-fold cross-validation, DenseNet121 achieved slightly superior overall performance compared with ResNet34, with higher validation accuracy (80% vs. 77%), precision (77% vs. 74%), recall (76% vs. 73%), and macro-AUC (0.933 vs. 0.916; *p* < 0.05). Per-class analysis showed that DenseNet121 performed best in the Decrease and Stop categories, while ResNet34 achieved marginally higher recall in the rare Increase class. Confusion matrix and ROC analyses confirmed that DenseNet121 produced fewer misclassifications and higher AUCs, indicating stronger discriminative ability and more consistent generalization across categories.

Ablation experiments demonstrated that the best performance was achieved with dropout layers, no pretrained weights, and a batch size of 32. Any modification to this configuration, such as using pretrained ImageNet weights, smaller batch size, or removing dropout, resulted in lower accuracy and recall, particularly for the Increase class. These results highlight the robustness of DenseNet121 and the effectiveness of the chosen training strategy for predicting post-brachytherapy response patterns in uveal melanoma.

This model provides a potential clinical decision-support tool by predicting the tumor response pattern before plaque brachytherapy using baseline ultrasound images. Such predictions could help ophthalmologists personalize follow-up strategies, for instance, by increasing surveillance frequency or considering adjunctive therapy in patients predicted to show Increase or Other patterns. In contrast, those predicted to respond with Decrease may follow standard follow-up intervals. In a practical workflow, a probability confidence threshold (e.g., ≥ 0.8) could be used to trigger closer monitoring; however, this threshold should be prospectively validated. Overall, the integration of AI-based predictive tools into ocular oncology practice may enhance individualized care, optimize resource allocation, and improve early detection of treatment failure.

This work has several limitations, including the use of a relatively small dataset, data acquired from a single center, and the absence of external validation. The limited dataset size restricts the model’s ability to generalize across broader populations and imaging settings. Data scarcity is a common challenge in medical imaging, particularly for rare conditions such as choroidal melanoma, and is further exacerbated by privacy concerns and limited access to annotated datasets. Collaborative multicenter studies and the development of shared databases are critical next steps to overcome these limitations and support model robustness. Although data augmentation and oversampling helped mitigate the severe imbalance between response classes, particularly the Increase category (4.8% of the dataset), this approach cannot fully reproduce the diversity of real clinical data. Therefore, while the model achieved high discriminative performance even for this rare class, its generalization to broader populations should be interpreted with caution. Clinically, however, even preliminary detection of rare post-brachytherapy progression cases is valuable, as it may prompt earlier ophthalmologic reassessment. Future studies with larger and multi-center cohorts are required to validate the robustness of the model in detecting such infrequent outcomes.

Furthermore, this study used 2D JPEG ultrasound images, which lack the full spatial and intensity information available in high-resolution 3D imaging. Future research should explore training with full-resolution or 3D ultrasound data, enabling the capture of richer spatial features and potentially improving diagnostic accuracy.

Beyond CNN-based models, future research could investigate the use of vision transformers, which have shown promise in capturing global context and improving performance in various medical imaging tasks. Moreover, the incorporation of explainable AI (XAI) methods, such as saliency maps, Grad-CAM, or concept-based attribution, would provide transparency into model decisions and foster trust among clinicians. Combining imaging data with clinical variables or biomarkers through multimodal learning approaches could also enhance model performance and lead to more personalized predictions.

## Conclusions

This study marks an attempt to develop a CNN-based model, using DenseNet121, to predict response patterns of UM following brachytherapy based on pre-treatment US images. The model successfully classified responses into four distinct categories: Increase, Decrease, Stop, and Other, achieving the highest accuracy in predicting the patterns. DenseNet121 demonstrated balanced performance across all classes, highlighting its potential as a tool for early treatment response assessment and personalized treatment planning. As the first of its kind, this approach lays the foundation for future advancements, where larger, multi-center datasets could further enhance model performance, enabling more robust and generalized predictions.

## Data Availability

The datasets used and analyzed during the current study will be available from the corresponding author on reasonable request.

## Abbreviations

AI: Artificial intelligence
ANN: Artificial neural networks
AUC: Area under the curve
CI: Confidence intervals
CNN: Convolutional neural network
CV: Cross validation
DL: Deep learning
DNS: Dysplastic nevus syndrome
FC: Fully connected
FN: False negative
FNAB: Fine needle aspiration biopsy
FPh: Fundus photography
FPR: False Positive Rate
LBD: Largest basal diameter
ML: Machine learning
MRI: Magnetic resonance images
NCI: National cancer institute
OCT: Optical Coherent Tomography
PCLs: Pigmented choroidal lesions
PCA: Principal component analysis
ROC: Receiver operating characteristic
SD: Standard deviation
SGD: Stochastic gradient descent
TN: True negative
TP: True positive
TTT: Transpupillary thermotherapy
UM: Uveal melanoma
US: Ultrasound
